# Fishing Cat Scats as a Biomonitoring Tool for Toxic Heavy Metal Contamination in Aquatic Ecosystems

**DOI:** 10.3390/toxics11020173

**Published:** 2023-02-13

**Authors:** Thirupati Lakshmi Harika, Khalid A. Al-Ghanim, Mian Nadeem Riaz, Kaliyamoorthy Krishnappa, Jeganathan Pandiyan, Marimuthu Govindarajan

**Affiliations:** 1 Department of Zoology and Wildlife Biology, A.V.C. College, Mannampandal, Mayiladuthurai 609 305, Tamil Nadu, India; 2 Department of Zoology, College of Science, King Saud University, Riyadh 11451, Saudi Arabia; 3 2476 TAMU, Texas A&M University, College Station, TX 77843, USA; 4 Unit of Mycology and Parasitology, Department of Zoology, Annamalai University, Annamalainagar 608 002, Tamil Nadu, India; 5 Unit of Natural Products and Nanotechnology, Department of Zoology, Government College for Women (Autonomous), Kumbakonam 612 001, Tamil Nadu, India

**Keywords:** heavy metal, toxicity, scats, prey species, mangrove forests, carnivores, conservation

## Abstract

Mangrove forest is one of the productive ecosystems that provide essential habitats for various fauna as breeding and feeding drives. However, heavy metal pollution in the mangrove forest has led to severe health problems for several aquatic species. Biomonitoring of metals using a nondestructive method is an emerging technique. Scats of the fishing cat (*Prionailurus viverrinus*) were collected from five locations in the Godavari estuary mangrove habitats, Coringa Wildlife Sanctuary, Andhra Pradesh, India, to determine the level of various metals. An opportunistic method was applied to collect scats in the mangrove forest. Six scat samples were collected from each of the sampling sites. The following prey species, such as crabs, fishes, birds, rodents, plants, plastics, and unidentifiable prey matters, were found in the scats. Select metals, such as chromium (Cr), copper (Cu), and lead (Pb) were analyzed from the scats of the fishing cat since they intensively influence the physiology and behavior of top predators. The concentration of Cu in fishing cat scats was higher than the other two metals assessed. Metals showed statistically substantial variation across locations (*p* < 0.05). According to the current study, heavy metals may significantly threaten the fishing cat in the Coringa Wildlife Sanctuary. The fishing cat is a vulnerable species in accordance with the ICUN categories. Due to pollution and other human pressures, the fishing cat may soon be categorized as a threatened or endangered species; the research advises that authorities should prioritize the protection of the vulnerable species of the fishing cat from the Coringa Wildlife Sanctuary, Andhra Pradesh, India.

## 1. Introduction

Metals are a class of non-biodegradable contaminants that cause extensive ecological damage by accumulating in aquatic ecosystems and posing substantial health risks to various organisms [1]. Evaluating heavy metals in the aquatic environment is crucial to comprehend the toxicity of metals and their effect on aquatic life. Countless animal species rely on mangrove forests for sustenance and shelter, making them an essential aquatic environment. Mangrove forests have been shown to play an important role in the lives of many animal species [2]. The eastern coast of southern India is home to one of India’s biggest mangrove forests, the Godavari forest. The Godavari mangrove forest has major ecological and biological functions in its estuary locations [3]. Due to its importance as a marine ecosystem, the Godavari mangroves have been declared as a “Coringa Wildlife Sanctuary.”

The Godavari mangrove is home to a variety of fishes, rats, and other prey species, and the dominant carnivore predator in this ecosystem is the *Prionailurus viverrinus*. The fishing cat *Prionailurus viverrinus* heavily uses the estuary mangrove forests of Godavari as a food source and a place to raise their young [4]. However, the decline of *Prionailurus viverrinus* numbers and their location remain as mysteries [5]. Moreover, the research mentioned little about the population and current location of the fishing cat, since very little has been learned about their status in the sanctuary [6]. Indeed, the fishing cat faces major problems owing to changes in the terrain, loss of their habitat, poisoning, overfishing, poaching for fur and road kill, and other aquafarm practices near their feeding and breeding habitats [7,8]. Meanwhile, a 2016 population study conducted by the International Union for Conservation of Nature (IUCN) [6] classified the fishing cat (*Prionailurus viverrinus*) as a vulnerable category.

The present research attempted to assess the select heavy metals, such as Cr, Cu, and Pb. Since the fishing cat is a protected and scheduled animal (Indian Wildlife Act, 1974), the assessment of metals has been carried out using scat samples. Globally, the biomonitoring of metals using a nondestructive method is very popular and significantly appreciated for the sake of the conservation and management of wildlife [1]. Indeed, levels of various heavy metals have been reported from the Godavari river [9] and the Godavari mangroves [10]. No research has been conducted on evaluating the metal content of the Godavari mangrove-dwelling fishing cat, which is regarded as a top predator species of the region [4,6,11]. A study envisaged that the top predators could be at risk due to heavy metal poisoning [11]. Nevertheless, chromium (Cr), copper (Cu), and lead (Pb) are known to have devastating effects on top predators in an ecosystem [12]. Therefore, the current study focused on the Cr, Cu, and Pb metals in the fishing cat, since these metals are predominantly biomagnified through the food chain of an aquatic ecosystem [9,10].

## 2. Materials and Methods

### 2.1. *Study Area*

**Figure 1:**
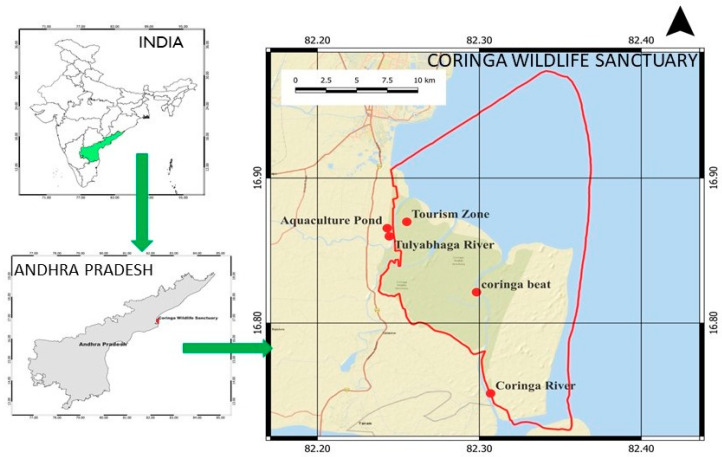
Map showing the five different study sites in the Coringa Wildlife Sanctuary, Andhra Pradesh, India. [Site 1: Tourism zone, Site 2: Coringa river, Site 3: Tulyabhaga river, Site 4: Coringa beat, Site 5: Aquaculture pond].

The Godavari mangroves are the second-largest mangrove forest located on the east coast of southern India. The forest size of 332.6 km^2^ makes it an ideal home and breeding ground for a wide variety of species (Figure 1). Coringa Wildlife Sanctuary, with a total area of 235.7 km^2^, was established in the Godavari mangroves. A total of 277 benthic creatures were recorded, including 35 mangrove species, 615 species of finfish, 269 species of birds, 26 species of reptiles, and 18 species of terrestrial animals [13]. The Godavari mangrove forest receives water from the Godavari river, which is one of the longest rivers in India. The river passes through various states and terminates on the east coast of southern India. Several small- to large-scale industries, factories, distilleries, granite factories, inosilicate mineral companies, fertilizer companies, etc., are located along the river basin, which are the major sources of metals in the Godavari river and Mangrove forests. In addition, aquaculture farms and other domestic effluents release wastewater into the Godavari mangrove forests [9,10].

In the current study, five different foraging sites of fishing cats were selected, i.e., Site 1: Tourism zone, Site 2: Coringa river, Site 3: Tulyabhaga river, Site 4: Coringa beat, Site 5: Aquaculture pond for the collection of scats of the fishing cat (Figure 1). The selection of these sites was based on the availability and abundance of scats (old and fresh). In addition, personal inquiries were conducted with the village people living adjacent to the mangrove forest. We have enquired about the presence or sighting of the fishing cat and recorded their views, and the sites were finalized to collect the scats for the present study. For each site, six scat samples were selected. The physical structure, age of the scat (days), and pugmark variations were used to differentiate among the different individuals of fishing cats.

### 2.2. Collection of Scats of Fishing Cat

**Figure 2:**
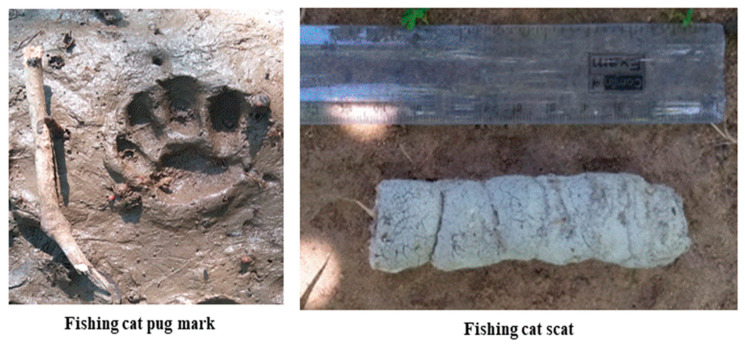
Pugmark and scat of the fishing cat *Prionailurus viverrinus*, Coringa Wildlife Sanctuary, India.

The scat samples of the fishing cat (*Prionailurus viverrinus*) were collected from five different sites (Figure 2) using an opportunistic method [14] from March to June 2022. The opportunistic method is conventional, in which the scats were collected from five distinct sites that the fishing cat used as a feeding area. For each site, six scats were collected for the study. The collected scat samples were wrapped in butter paper and transported to the lab in Ziplock covers for further metal analysis.

### 2.3. Preparation of Scat Samples for Metal Analysis

**Figure 3:**
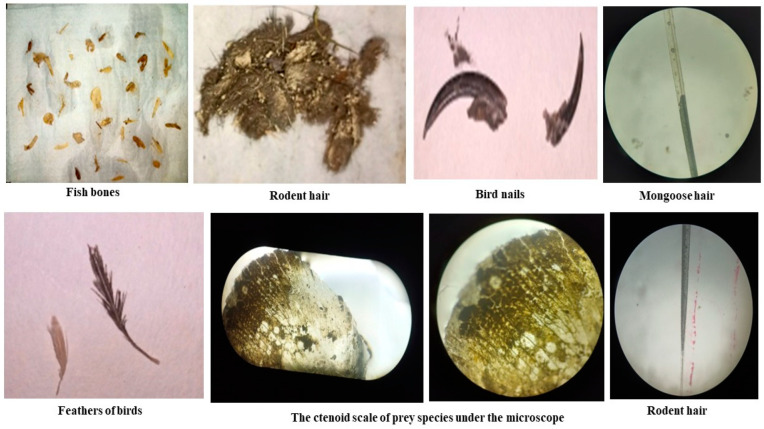
Prey matters of fishing cats found from their scat samples at five different sites of the study area.

Scat samples of the fishing cat were soaked in double distilled water for 24 h. Then, the soaked scats were sieved using a 1 mm sieve and the excess water was removed from the soaked samples using Whatman filter paper. Next, the dehydrated scat samples were transferred to a Petri dish and were focused under a light microscope to identify the prey species present in the scat sample of the fishing cat [15]. Finally, the prey matters, such as fishbone, rodent hair, bird nails, mongoose hair, bird feathers, and the Ctenoid scale of fishes, were found and left for further analysis (Figure 3).

### 2.4. Digestion of Scats of Fishing Cat

The scats’ length, breadth, and weight were measured and dried using a hot sterilizer for 24 h at 105 °C. The scat remnants were homogenized and weighed using an electronic balance [16]. Five grams of the homogenized dry scat was digested using a microwave digester (Milestone, MLS 1200) with 10 ml of nitric acid (69% grade) for 10 min, and 1 ml of perchloric acid (70% grade) for 5 min [17]. The digested samples were left in a deep freezer until metal analysis, such as Cr, Cu, and Pb, using atomic absorption spectroscopy (AAS) [18,19].

### 2.5. Quality Control

The steady state of the equipment can be determined by injecting each of the three QC samples. To ascertain the precision of the results, three blank, standard and samples were used for each set of the metal analysis from the scat samples. Standard curves were also prepared for Cr, Cu, and Pb at 05, 1.0, 2.0, 5.0, and 10 ppm. The AAS is fixed to a level of zero for each sample with a blank. The metal results are reported as ppm [1,19].

### 2.6. Data Analysis

Arithmetic mean and standard error were measured to relate the results of heavy metal contamination. After normality was tested (Shapiro–Wilk), a one-way analysis of variance (ANOVA) was computed to validate the difference in metal concentration among the sites. The analysis was performed using SPSS 25.0, and the level of significance is *p* < 0.05. The results are inferred by Sokal and Rohl [20].

## 3. Results

A variety of crustaceans, fishes, birds, rodents, plants, plastics, and unidentifiable prey items were discovered in fishing cat scats (Table 1). The contents of their scat showed various types of consumed prey items, such as shells and carapaces from crustaceans; scales, fins, and bones from fish; feathers, bones, and claws from birds; hair, bone, teeth, and nails from rodents; leaves, grass, prickly spikes, and heartwood from plants; and plastics of varying sizes (Table 1). The one-way ANOVA revealed that the quantity and composition of prey matters showed significant differences among the sites studied (*p* < 0.05).

**Figure 4:**
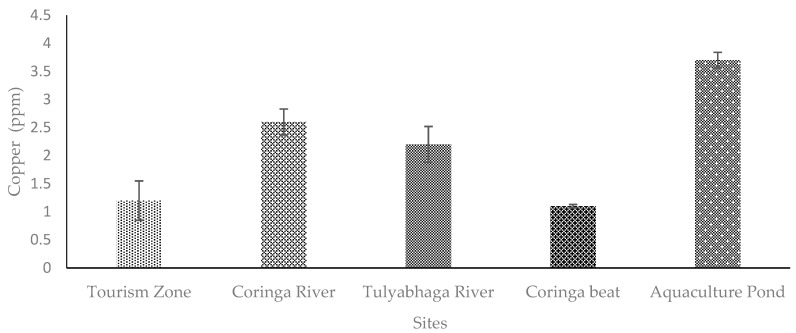
Level of Cu examined in fishing cat scats from different sites of Coringa Wildlife Sanctuary, Andhra Pradesh, India. [Tourism zone (Site 1), Coringa river (Site 2), Tulyabhaga river (Site 3), Coringa beat (Site 4), and Aquaculture pond (Site 5)]. [Bar indicates mean and the line indicates SE; *n* = 6].

**Figure 5:**
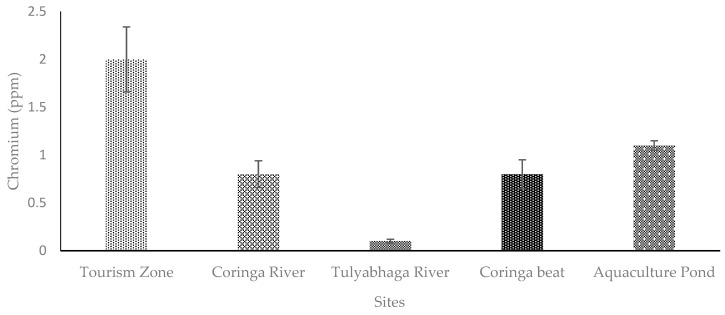
Level of Cr examined in fishing cat scats from different sites of Coringa Wildlife Sanctuary, Andhra Pradesh, India. [Bar indicates mean and line indicates SE; *n* = 6].

**Figure 6:**
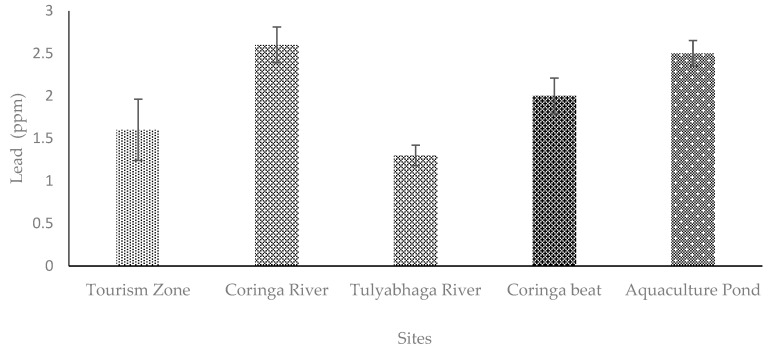
Level of Pb examined in fishing cat scats from different sites of Coringa Wildlife Sanctuary, Andhra Pradesh, India. [Bar indicates mean and line indicates SE; *n* = 6].

## 4. Discussion

The aquatic environment provides sustainable homes for diverse species of fauna and flora throughout their life cycle; however, the majority of the aquatic ecosystem is polluted and damaged by various forms of pollution, with heavy metals being the most damaging [21]. According to research, heavy metals are significantly more harmful than other toxic elements in the aquatic ecosystem [1]. Due to metal contamination, severe detrimental effects have been observed on several aquatic animal species, particularly top predators [22]. The research analyzed the Cu, Cr, and Pb levels in the feces of the fishing cat, which is the main predator in the Coringa Wildlife Sanctuary [4]. Researchers discovered that mammalian feces are the most accurate source for assessing metal concentrations [23]. In fact, heavy metals in carnivores were evaluated utilizing nondestructive procedures in the interest of their welfare and management [24,25]. Similar nondestructive approaches were used to analyze certain metals in other species, such as feathers and egg shells in birds [1,12,17,18,19]. Cu, Cr, and Pb have noteworthy physiological and behavioral effects on a variety of animal species [26,27,28,29,30]. Copper, chromium, and lead were found in higher amounts in the feces of the fishing cat from the Coringa Wildlife Sanctuary than in the feces of other mammal species tested elsewhere in the globe, as determined by the present research.

### 4.1. Copper (Cu)

Copper is a fundamental nutrient, which is needed by all living things in order to survive. Moreover, copper has distinct physiological activities, particularly regarding the structure and function of amino acids [31]. In contrast, quarrying, melting, granulating, agricultural activities, and the improper disposal of both liquid and solid wastes all contribute to the presence of copper in aquatic environments. Agro-farming operations, biogeochemical cycles, and industrial effluents contribute to Cu reabsorption in aquatic ecosystems [17,26]. The mangrove ecosystem of Coringa Wildlife Sanctuary is threatened by the influx of contaminated water from nearby rivers, canals, aquafarms, factories, and businesses [10]. The present research revealed a higher concentration of Cu in fishing cat scats obtained from the Coringa river, the Tulyabhaga river, the Coringa beat, and Aquaculture pond (Figure 4, Figure 5 and Figure 6). The carnivorous fishing cat dines mostly on fish, birds, rodents, and crabs. Indeed, the Cu is stored in higher concentrations in smaller animals, such as rats, while marine fishes, shorebirds and crustaceans are known to be exposed to a larger amount of Cu [32,33]. Nevertheless, studies indicated that the Godavari river water and the soil sediment of the Coringa mangrove had a greater level of Cu [9,10]. Therefore, it is possible that the fishing cat showed greater level of Cu, which is possibly due to their diet preferences in the mangrove forest.

### 4.2. Chromium (Cr)

The major sources of Cr in aquatic environments are through the biological factors, petroleum coal, chromium factories, fertilizer companies, metal plate, and leather industries etc. Another source of Cr in wetland environments is by anthropogenic activities in the estuary layers [34]. A study revealed that the Cr is enhanced due to the deposition of wastewater from homes and factories [35]. According to the research, the Cr levels were highest in the fishing cat scat at Site 1 (Tourist zone) of the Coringa Wildlife Sanctuary. Tourists that flock to the sanctuary’s most popular attractions may have taken items made from chromium and added them to this existing element. In addition, fish, crabs, and birds have higher Cr [36]. Moreover, the current study’s research on the food composition of the fishing cat revealed that it forages on fish, crabs, birds, and vegetation (Table 1). As a result, their foraging strategies and choice of prey affect the amount of Cr in fishing cat scats. Coringa Wildlife Sanctuary has been shown to have significant levels (*p* < 0.05) of metal contamination, particularly Cr [9,10]. Therefore, this might be the reason that the level of Cr was greater in fishing cat scats.

### 4.3. Lead (Pb)

Pb has several protein functions and is involved in cellular metabolism [31]. Pb in aquatic environments comes from human activities, such as sewage disposal, coal burning, paint production, automobile production, leather processing, oil refining, etc. [37,38]. The present investigation was conducted at the Coringa Wildlife Sanctuary, where water is piped in from the Godavari river and its tributaries through a network of canals that pass through several human-populated areas. Coringa mangrove forest water, sediment, and the Godavari river water have been reported to contain traces of lead [9,10]. Toxic metals may leach out of contaminated water as well as sediment and contaminate aquatic species, including crustaceans, fish, birds, rodents, and plants [39,40,41,42]. The Pb is higher in rats, since they consume a wide variety of plant and animal products, as observed by Way and Schroder [43]. The current study found that the level of Pb in fishing cat scat was in greater concentrations. The diet composition of the fishing cat scat showed that the cat feeds on crustaceans, fishes, birds, and rodents (Table 1). Therefore, the study suggested the level of Pb in the fishing cat through their diet and feeding habits. According to a previous study [44], lead poisoning is a major problem among carnivorous animals. Studies have shown that Pb may have devastating effects on a variety of animal physiological systems [45,46].

Nevertheless, the concentrations of Cr, Cu, and Pb were greater in the fishing cat than the other studied mammals, such as tiger, wild boar, wild cats, deer, rodents, rabbits, nilgai, porcupine, wild cats, etc., [22,23,24,28,43], which is one of the remarkable findings of the study. In addition, the study revealed that the metal concentrations varied significantly (*p* < 0.05) among the five different sites (Figure 4, Figure 5 and Figure 6). The variations in the level of metals among the sites might be due to the composition of prey species, the size and weight of the prey, and other ecological factors. The concentration of Cr, Cu, and Pb in the fishing cat is above the threshold level, and thus could affect the physiology and behavior of the fishing cats. Studies have stated that severe physiological and behavioral changes have been reported due to the metal’s poison in animals [47,48].

## 5. Conclusions

The study explored the level of metals, such as Cr, Cu, and Pb, in the vulnerable fishing cat (*Prionailurus viverrinus*) using scat samples from their feeding and breeding sites of the sanctuary. The fishing cat is a top predator in the Coringa Wildlife Sanctuary and is regarded as an important or keystone species of the sanctuary. However, rigorous studies need to be carried out on the assessment of metals, including other toxic metals which are not covered in this study. Moreover, the metals from water, soil, and their prey species are essentially assessed to achieve knowledge on the pollution and quality of the sanctuary for better management of the vulnerable fishing cat. Furthermore, the authorities should frame guidelines on the discharges of various effluents in the Godavari river, regulations on tourism, and a proper protocol for the outlet of aquafarms for the management of the sanctuary and the wild cat, which purely depends on the sanctuary.

## Figures and Tables

**Table 1:** Prey species and their composition found in the scats of fishing cat, Coringa Wildlife Sanctuary, Andhra Pradesh, India from March to June 2022 (values are mean and SE; *n* = 6).

Prey Species (mg)	Prey Composition	Different Sites				
		Tourism Zone (*n* = 6)	Coringa River (*n* = 6)	Tulyabhaga River (*n* = 6)	Coringa Beat (*n* = 6)	Aquafarms (*n* = 6)
Crustaceans	Shells	3.3 ± 1.11	3.3 ± 0.99	0.3 ± 0.28	1.5 ± 0.61	3.5 ± 0.98
Fish	Scales	4.1 ± 0.73	3.1 ± 0.86	3.3 ± 0.95	5.4 ± 0.87	5.4 ± 1.07
	Fins	1.4 ± 0.68	2.1 ± 0.85	0.5 ± 0.36	3.1 ± 0.67	6.5 ± 1.54
	Bones	2.8 ± 0.71	1.8 ± 0.61	1.7 ± 0.68	2.1 ± 0.80	1.4 ± 0.07
Bird	Feathers	2.9 ± 0.80	2.4 ± 0.63	2.0 ± 0.71	1.7 ± 0.60	1.1 ± 0.90
	Bones	1.8 ± 0.71	1.7 ± 0.71	2.0 ± 0.86	0.5 ± 0.29	0
	Claws	1.1 ± 0.57	1.0 ± 0.43	1.2 ± 0.54	1.2 ± 0.62	4.5 ± 1.23
Rodent	Hair	5.2 ± 1.02	4.5 ± 1.10	4.0 ± 0.98	5.8 ± 0.63	1.4 ± 0.58
	Bone	1.9 ± 0.75	2.0 ± 0.80	1.3 ± 0.73	2.2 ± 0.62	0.1 ± 0.15
	Teeth	1.8 ± 0.80	3.4 ± 1.11	2.4 ± 1.16	1.8 ± 0.85	0
	Nails	1.2 ± 0.64	1.3 ± 0.66	0.7 ± 0.47	0.3 ± 0.27	3.5 ± 0.86
Plant	Leaves	2.3 ± 0.64	2.0 ± 0.84	1.4 ± 0.59	1.8 ± 0.62	1.7 ± 0.95
	Grass	4.1 ± 0.98	3.0 ± 0.97	3.9 ± 0.94	4.4 ± 0.81	0.3 ± 0.27
	Heartwood	0.5 ± 0.37	1.1 ± 0.61	1.2 ± 1.02	0.9 ± 0.59	0
	Thorny spikes	1.8 ± 0.63	1.4 ± 0.71	1.2 ± 0.69	1.0 ± 0.51	0
Unidentified	Fauna/floral crumbles	1.4 ± 0.82	1.9 ± 0.90	0.6 ± 0.40	1.0 ± 0.44	1.7 ± 0.60
Others	Plastics/polythene/nylon	61.0 ± 3.87	61.5 ± 2.49	61.3 ± 7.56	64.0 ± 2.13	66.9 ± 1.85
Digested weight	Filtered/purely soluble	0	0	9.9 ± 9.91	0	0
Total weight	Dry weight	4.1 ± 0.73	3.1 ± 0.86	3.3 ± 0.95	5.4 ± 0.87	3.5 ± 0.98

chromium (Cr), copper (Cu), and lead (Pb) were assessed from the scats of the fishing cat *Prionailurus viverrinus*. The Cr was greater (2 ± 0.39 ppm) in Site 1 (Tourism zone) than the other four sites examined (Figure 4). However, the Pb was higher (2.6 ± 0.12 ppm) in Site 2 (Coringa river) and Site 5 (Aquaculture pond; 3.7 ± 0.57 ppm) (Figure 5). The highest concentrations of Cu were noted in the Aquaculture sites (3.7 ± 0.14 ppm). The concentrations of Cr, Cu, and Pb levels differed significantly (*p* < 0.05) among the sites studied in fishing cat scats from the various sites of Coringa Wildlife Sanctuary (Figure 4, Figure 5 and Figure 6).

## Data Availability

Not applicable.
